# A molecular nanodevice for targeted degradation of mRNA during protein synthesis

**DOI:** 10.1038/srep20733

**Published:** 2016-02-09

**Authors:** Kyung-Ho Lee, Seung-Eui Min, Haseong Kim, Seung-Goo Lee, Dong-Myung Kim

**Affiliations:** 1 Department of Chemical Engineering and Applied Chemistry, Chungnam National University, Daejeon 305-764, Korea; 2 Synthetic Biology and Bioengineering Research Center, KRIBB, Daejeon 34141, Korea

## Abstract

RNase H is an endonuclease that catalyzes the cleavage of RNA. Because it only acts on RNA in RNA:DNA hybrids, RNase H can be used for targeted degradation of RNA when used in combination with antisense oligodeoxyribonucleotides (ASODNs) designed against a specific sequence of the target RNA. In this study, ASODN and RNase H were co-conjugated on magnetic nanoparticles. The resulting nanoparticles, having integrated functions of probing and processing target RNA, were able to remove target mRNA sequences more effectively than free ASODNs. The paramagnetic property of the nanoparticles also enabled timed engagement and disengagement of the RNA-degrading components in a given system, and these nanoparticles were able to be used for ON/OFF control of gene expression during cell-free protein synthesis reactions.

Integration of the unique properties of nanoparticles and biomolecules has been in the spotlight in recent years. Having comparable dimensions, nanoparticles and biomolecules such as proteins and DNA can be conjugated to form new entities with tailored functions^1^,^2^,^3^. From the view point of synthetic biology aimed at the rational and systematic design of biological systems that do not exist in nature, the use of nanoparticles is expected to expand our capability to manipulate and consolidate individual biological parts into new systems designed for specific purposes^4^,^5^,^6^. Upon combination with nanoparticles, biomolecules often exhibit unusual properties that are useful for their applications. For example, Seferos *et al*. reported that conjugation on nanoparticles enhanced the stability of oligodeoxynucleotides (ODN) against nonspecific nucleases^7^. In contrast, interestingly, ODN-functionalized nanoparticles selectively enhanced ribonuclease H (RNase H) activity^8^. ODN designed to hybridize with specific nucleotide sequences of mRNA (antisenseoligonucleotide, ASODN) can trigger targeted degradation of mRNA, mediated by RNase H^9^,^10^,^11^. ASODN probes target sequences of mRNA and guides RNase H to initiate degradation of ASODN-hybridized mRNA. Therefore, this combination of properties carries significant implications in the development of robust method for regulation of gene expression. In this study, to further improve the efficiency of targeted mRNA degradation, we designed magnetic nanoparticles co-functionalized with ASODN and RNase H. In this scheme (Fig. 1), ASODN on nanoparticles attracts target RNA through complementary hybridization, and the neighboring RNase H digests the hybridized RNA. Therefore, this design adds the function of target processing to the nanoparticles that probe target mRNA via conjugated ASODN. We explored the use of the nanoparticles as a switching device for the control of gene expression in a cell-free protein synthesis system. During the cell-free protein synthesis reaction programmed with superfolder green fluorescent protein (sfGFP) gene, protein synthesis was halted upon the addition of the nanoparticles carrying ASODN/RNase H against a selected sequence of sfGFP mRNA. Through the use of paramagnetic nanoparticles as the substrates for conjugation, ASODN/RNase H was able to be extracted from the reaction mixture with a magnet, resulting in an instant resumption of protein synthesis. As a consequence, alternating addition and removal of the nanoparticles allowed stop-and-go control of protein synthesis. Our results showed that functionalized nanoparticles serve as a versatile interface for the mix-and-match application of biological parts in synthetic biology studies, which can be used for the design, analysis and evaluation of genetic circuits.

## Results

### Design and selection of ASODNs against the sfGFP mRNA for the gene suppression in a cell-free protein synthesis system

**Figure 2:**
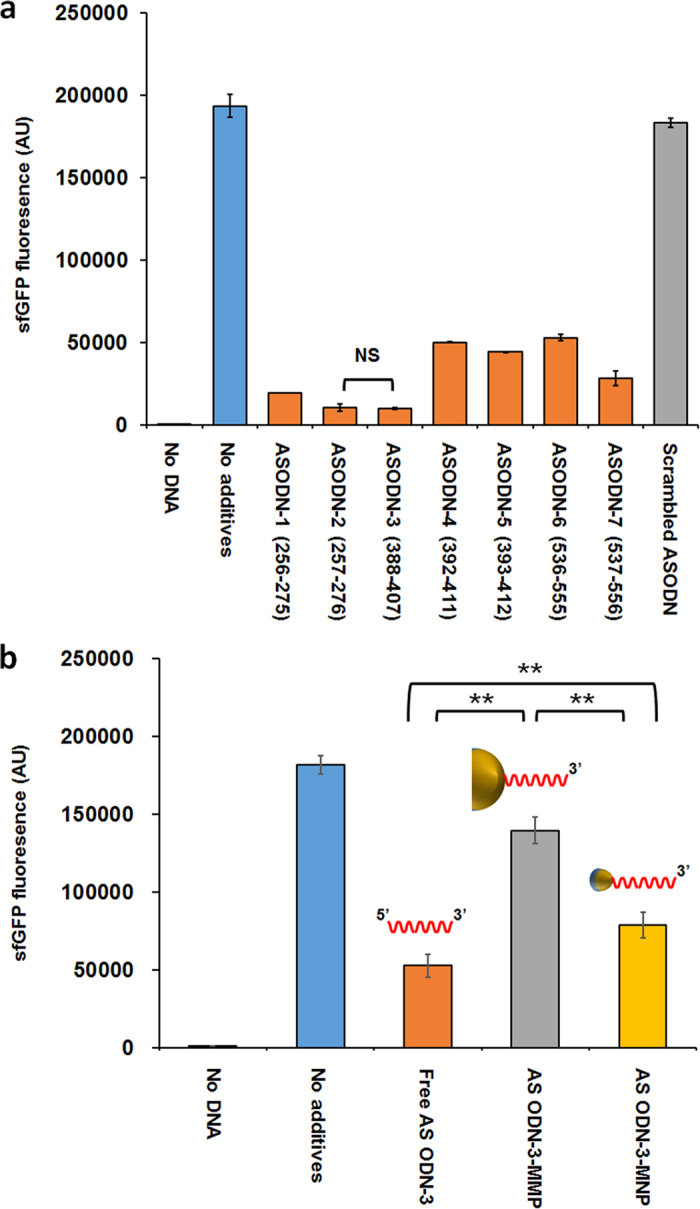
Suppression of sfGFP expression with free ASODNs or ASODN conjugated with magnetic particles. (**a**) ASODN-mediated suppression of gene expression during cell-free protein synthesis. Seven ASODNs targeting sfGFP mRNA were selected from the sequences designed by the Sfold program. Each of the ASODNs (ASODN-1 through ASODN-7, along with a scrambled ASODN as a control) was added to separate reaction mixtures for cell-free synthesis of sfGFP. sfGFP fluorescence was detected after the protein synthesis reaction (N = 4). (**b**) Suppression of sfGFP expression in the presence of free ASODN, ASODN-MMP or ASODN-MNP. ASODNs were conjugated on the particles at their 5′-end. Fluorescence of the synthesized sfGFP was detected after the protein synthesis reaction with free ASODN or conjugates (N = 3). N means the number of independent reaction samples. Error bars in the graphs represent standard deviations. NS, not significant.

For the ASODN-mediated control of gene expression during cell-free protein synthesis, we first designed seven ASODNs targeting different regions of the sfGFP mRNA using Sfold software (Supplementary Table 1)^12^,^13^. ASODNs were examined for their suppression efficiency of gene expression in a standard reaction mixture for cell-free protein synthesis programmed with pK7 sfGFP, which carries the sfGFP gene under the control of the T7 promoter. Because a crude cell extract was used in the present cell-free protein synthesis system, no additional RNase H was provided in the reaction mixture^14^. When determined by the relative fluorescent intensities of sfGFP cell-free synthesized in the presence of 60 μM ASODN, the suppression efficiencies of the designed ASODNs generally showed a good correlation with the ∆G values of the ASODN-mRNA hybrids (Supplementary Table 1 and Fig. 2a). Among the ASODNs examined, ASODN-2 and ASODN-3 had the highest suppression rate at the concentration used (94.4 ± 1.05% and 94.7 ± 0.2%, respectively; less than 0.01 of p-value in student’s t-test compared to other ASODNs). The difference between ASODN-2 and ASODN-3 was not significant (p-value = 0.638) and ASODN-3 was used for subsequent experiments.

### Effect of conjugational orientation of ASODNs and the nanoparticles on its antisense activity for gene suppression

**Figure 3:**
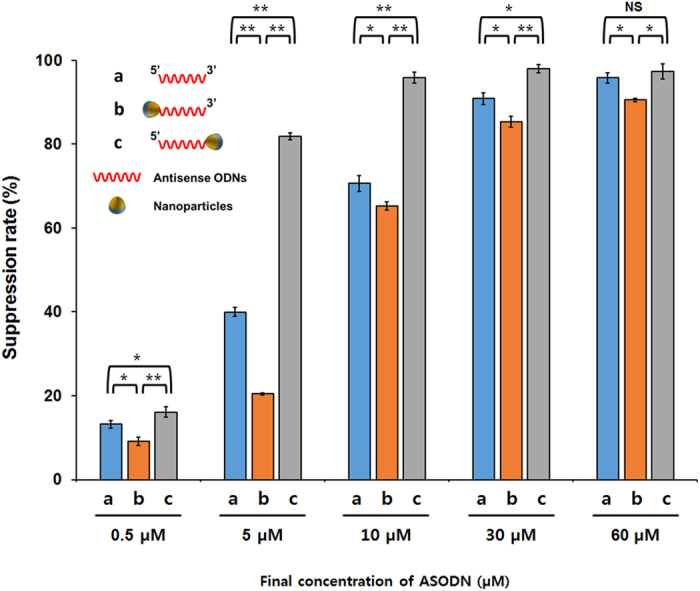
Suppression of gene expression in the presence of varying concentrations of free ASODN or MNP-conjugated ASODN. Suppression rate was determined by the ratio of the sfGFP fluorescence in the presence and absence of ASODS. (**a**) Free ASODN. (**b**) MNP conjugated with 5′-end of ASODN. (**c**) MNP conjugated with 3′-end of ASODN. Error bars in the graph indicate standard deviations from three independent reaction samples. *p-value < 0.05. **p-value < 0.01. NS, not significant.

The cell extract used in this study was derived from the crude lysate of *E. coli* cells^15^ and thus contained substantial amounts of exonucleases^16^,^17^,^18^,^19^,^20^, which should degrade ASODN added to the reaction mixture and reduce the suppression efficiency. Therefore, in an attempt to extend the half-life of ASODN by physically blocking an end of ASODN, 5′-amine modified ASODN was immobilized on the surface of magnetic microparticles (MMP; average diameter: ~1 μm) or nanoparticles (MNP; average diameter: ~50 nm). Validity of the conjugation chemistry was confirmed by confocal microscopic images of the microparticles before and after conjugation with 5′-carboxyfluorescein (FAM)-modified ASODN (Supplementary Fig. 1). However, contrary to our expectation, both MMP-conjugated and MNP-conjugated ASODN produced lower suppression of protein synthesis compared to free ASODN of same concentration. At the concentration of 10 μM, suppression efficiencies for free ASODN, MMP-conjugated ASODN and MNP-conjugated ASODN were 75.26 ± 3.16%, 22.18 ± 3.98% and 56.07 ± 3.67%, respectively (Fig. 2b). Lower suppression efficiency of particle-conjugated ASODNs seemed to be due to their decreased mobility, which can explain why MNP-conjugated ASODN showed significantly higher suppression efficiency than did MMP-conjugated ASODN. In addition, it was found that both the micro- and nanoparticles tend to aggregate after conjugation with ASODN (Supplementary Fig. 1 and Supplementary Fig. 2), which can also reduce the number of ASODN available for hybridization with target mRNA. On the basis of these results, we concluded that the possible stabilizing effect of particle conjugation was offset by the reduced mobility of the conjugated ASODNs. During this study, we made a fortuitous discovery that the orientation of ASODN conjugation was a crucial factor affecting the stability of ASODN and suppression of gene expression. As seen in Fig. 3, both 5′-conjugated and 3′-conjugated ASODN showed concentration-dependent suppression of translation of sfGFP. However, compared to 5′-conjugated, 3′-conjugated ASODN suppressed the translation reaction far more efficiently. At 10 μM, 3′-conjugated ASODN on MNP produced almost complete suppression of translation (97.83 ± 1.32%) compared to 65.22 ± 0.98% of suppression with 5′-conjugated ASODN (p-value < 0.01). It should be noted that the suppression efficiency of 3′-conjugated was even higher than that of free ASODN at the same concentration (72.31 ± 1.81%, p-value < 0.01). The enhanced suppression efficiency of the 3′-conjugated ASODN appeared to be associated with improved stability of the conjugated ASODN, which led us to speculate that the 3′-exonucleases were the dominant exo-DNases present in the cell extract. Differential stability of ASODN depending on the orientation of conjugation was also confirmed from the recycled use of the nanoparticle-conjugated ASODNs. After a round of cell-free synthesis reaction (3 h at 30 °C), magnetic nanoparticles were recovered using a neodymium magnet, washed with phosphate-buffered saline (PBS), and added back to a fresh reaction mixture for another round of protein synthesis reaction. While the suppression efficiency of the 5′-conjugated ASODN showed a sharp decrease with repeated use, 3′-conjugated ASODN showed a far more consistent suppression efficiency. Less than 20~30% decrease in the suppression efficiency was observed per reuse step (Supplementary Fig. 3). On the basis of these results, 3′-conjugated ASODS were used in the subsequent experiments.

### Effective suppression of target gene using the nanoparticles with ASODN and RNase H in a cell-free protein synthesis

**Figure 4:**
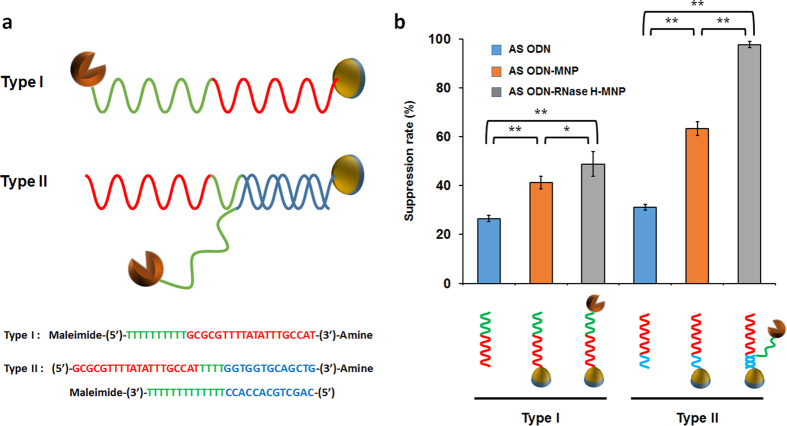
Design and comparison of molecular nanodevices consisting of ASODN, RNase H and magnetic nanoparticles. (**a**) Schematic of the two designed MNP conjugates with ASODN and RNase H. Red letters, ASODN regions; green letters, linker regions; blue letters, complementary regions. (**b**) Comparison of suppression rates of the two designed nanodevices during cell-free protein synthesis of sfGFP. Final concentration of ASODN was set at 2 μM and approximately same concentrations of RNase H was conjugated on MNPs. Error bars in the graph indicate standard deviations from three independent reaction samples. *p-value < 0.05. **p-value < 0.01.

It is commonly observed in cells that functionally related biological components form dynamic and/or static complexes. As shown in glycolysis and TCA cycle, enzymes driving the major cellular process are often found as complexes of multiple enzymes rather than stand-alone individual enzymes^21^. The benefits of complexed enzymes are obvious: since the intermediate molecules are delivered to the next enzyme in a channeled flux, the entire process rate can be enhanced compared to the reactions catalyzed by individual enzymes relying on random diffusion of substrates. Likewise, in the design of synthetic biological tools, physical integration of participating components will bring about enhanced efficiency of the intended biological processes. We applied this concept to ASODN-mediated degradation of mRNA. For the purpose of cooperative degradation of target mRNA, ASODN (probing unit) and RNase H (processing unit) were conjugated on the same magnetic nanoparticles (carrier unit). Two types of DNA linkers were used to bring ASODN and RNase H in close proximity on the nanoparticles in different configurations (Fig. 4a). In Type I configuration, ASODN was used as the front part of a single-stranded DNA linker, whose end was conjugated with RNase H. In Type II configuration, RNase H-conjugated DNA and ASODN-containing DNA were prepared separately and assembled through their complementary hybridization.

When prepared as described in ‘Methods’ section, the molar ratio of ASODN and RNase H conjugated on magnetic nanoparticles was approximately 1:1 in both configurations, indicating that the conjugation process was completed as designed. Comparative experiments revealed that both configurations of the nanodevices showed a substantially higher suppression efficiency than did ASODN simply conjugated on the nanoparticle or free ASODN. With ASODN as low as 2 μM, more than 49.85 ± 5.43% suppression was obtained with Type I nanoparticles, compared to 40.23 ± 2.61% suppression with nanoparticles conjugated to ASODN alone. The effect of Type II conjugation was far more dramatic, and almost complete suppression (97.29 ± 0.3%) of translation was achieved with the same concentration (2 μM) of ASODN (Fig. 4b). The higher suppression efficiency of Type II nanoparticles was thought to be due to the fact that less bending of the linker DNA is required for the conjugated RNase H to access the ASODN-hybridized mRNA.

### ON/OFF control of target gene using the molecular nanodevice with ASODNs and RNase H during cell-free protein synthesis

**Figure 5:**
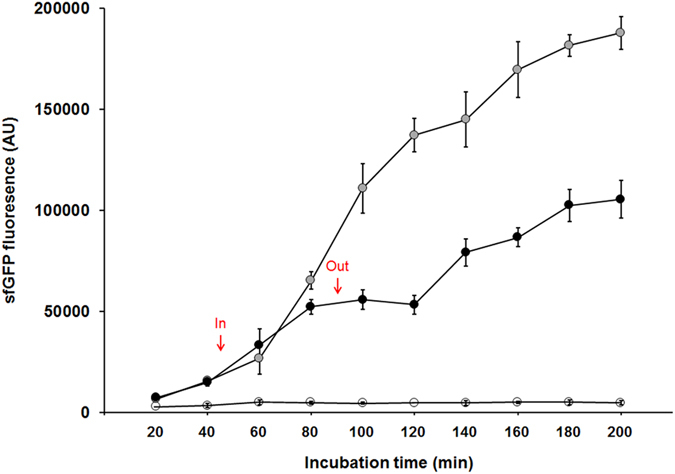
ON/OFF control of gene expression using ASODN/RNase H-MNP conjugate (Type II). Cell-free protein synthesis mixtures were incubated at 30 °C in the absence (grey circles) or presence (blank circles) of the ASODN/RNase H-MNP conjugate. In a parallel experiment, ASODN/RNase H-MNP conjugate was added to the reaction mixture at 45 min and removed at 90 min (black circles). Fluorescence of cell-free synthesized sfGFP was measured every 20 min. Error bars in the graph indicate standard deviations from three independent reaction samples.

Type II hybrid nanoparticles were then applied for on-demand regulation of gene expression in a cell-free protein synthesis system. As demonstrated in our previous studies, a cell-free protein synthesis system provides an attractive platform in synthetic biology efforts in that it enables highly flexible and designable control of gene expression^14^,^22^. Taking advantage of the simple removability of the magnetic nanoparticles from the reaction mixture, the ASODN and RNase H on the Type II hybrid nanoparticles were investigated as a movable agent for reversible ON/OFF control of suppression of gene expression in cell-free protein synthesis reactions. As described in the above section, ASODN was conjugated through its 3′-end amine group. The OFF control of gene expression was achieved by adding the hybrid nanoparticles with ASODN at a final concentration of 2 μM at 45 min in the cell-free protein synthesis reaction. As shown in Fig. 5, the addition of MNP-conjugated ASODN instantly decreased the rate of protein accumulation in the reaction mixture. After the addition of Type II hybrid nanoparticles at the indicated time point (45 min) during cell-free synthesis of sfGFP, compared to a control reaction without any ASODN, far less accumulation of protein was observed at the time point of 80 min (p-value = 0.016). Next, the nanoparticles were removed at 90 min by using a magnet to examine if cell-free protein synthesis of sfGFP could be resumed. As expected, sfGFP protein synthesis resumed gradually upon the removal of the nanoparticles. As a result, alternating addition and removal of the hybrid nanoparticles enabled stop-and-go control of gene expression.

## Discussion

Oligonucleotides are exploited to regulate the molecular process of gene expression through binding to target mRNA^23^,^24^. At present, three approaches are available for using oligonucleotides to interfere the translation of mRNA into proteins: RNA interference (RNAi), ASODN and steric-blocking oligonucleotides^25^. Among those, modulation of gene expression by RNAi and ASODN requires cooperation with enzymes. Therefore, measures to co-localize oligonucleotides with hydrolytic enzymes will increase the efficiency of target degradation. In this study, we designed a molecular nanodevice where multiple functions required for selective modulation of gene expression were integrated. This self-sufficient molecular nanodevice consisted of the units for probing (ASODN) and processing (RNase H) target mRNAs on a removable solid platform (magnetic nanoparticles). During the investigation on the design of this nanostructure, different factors were found to be crucial for the hybrid nanoparticles to degrade mRNA targets efficiently. First, the orientation of conjugation substantially affected the suppression efficiency of gene expression of the conjugated ASODNs. Most likely through the higher stability against the exonucleases in the cell extract, 3′-conjugated ASODN showed far more efficient suppression of gene expression than did the same ASODN conjugated at the 5′-end. The efficiency of the ASODN-mediated mRNA degradation was also dramatically affected by the configuration to bring the ASODN and RNase H in proximity as demonstrated by the comparison of Type I and Type II nanoparticles. This result implies that the efficiency of targeted degradation of mRNA can be further improved if the orientation of RNase H immobilization is controlled towards the ASODN. We are currently working on site-specific conjugation of RNase H on the linker sequence for this purpose. The magnetic nanoparticles designed in this study not only provided the surface for co-conjugation of the two biological units, but also enabled easy extraction of the ASODN/RNase H pair out of the whole reaction system, when the degradation process needed to be halted. We thus could use these nanoparticles for timed engagement and disengagement of the mRNA-degrading machinery for on-demand ON/OFF control of gene expression.

Considering that various nanoparticles are being actively sought as a promising means for intracellular delivery of biological materials^26^,^27^, we expect that the use of described molecular device can potentially be extended for the control of gene expression inside cells.

## Methods

### Materials

Dynabeads^®^ MyOne™ microbeads (average diameter of 1 μm) and FluidMAG-nanoparicles (average diameter of 50 nm) were purchased from Invitrogen (Carlsbad, CA, USA) and Chemicell (Berlin, Germany), respectively. RNase H was from Takara Bio Inc (Otsu, Japan). Nucleotide triphosphates, creatine phosphate (CP), and creatine kinase (CK) were purchased from Roche Applied Science (Indianapolis, IN, USA). All other chemical reagents were purchased from Sigma (St Louis, MO, USA) and used without further purification. The S12 extract was prepared from the strain BL21-Star^TM^ (DE3) (Invitrogen) following previously described procedures^15^.

### Targeted degradation of mRNA using the self-sufficient hybrid nanoparticles

Sfold software was used for thedesign of ASODNs^13^. Initially, seven 20-mer ASODNs (sfGFP256-275, sfGFP257-276, sfGFP388-407, sfGFP392-411, sfGFP393-412, sfGFP536-555 and sfGFP537-556) that targeted the sfGFP mRNA with varying binding site disruption energy were selected, and their suppression efficiency of gene expression was evaluated during cell-free protein expression of sfGFP. The ASODN sequences and mRNA targeting sites are listed in Supplementary Table 1.

### Cell-free protein synthesis

In the experiments for ON/OFF control of gene expression in a cell-free protein synthesis system, the sfGFP gene was PCR-amplified from pY71sfGFP^28^ with primers containing NdeI and SalI sites. After restriction digestion with corresponding restriction enzymes, amplicons were cloned into pK7 plasmids between the T7 promoter and the T7 terminator as previously described^29^. The standard reaction mixture used for the cell-free protein synthesis consisted of the following components in a final volume of 15 μL: 57 mM Hepes–KOH (pH 8.2), 1.2 mM ATP, 0.85 mM each of CTP, GTP, and UTP, 2 mM DTT, 0.64 mM cAMP, 90 mM potassium glutamate, 80 mM ammonium acetate, 12 mM magnesium acetate, 34 mg/mL l-5-formyl-5,6,7,8-tetrahydrofolic acid (folinic acid), 1.5 mM each of 20 amino acids, 2% PEG (8000), 67 mM creatine phosphate (CP), 3.2 mg/mL creatine kinase, and 4 μL of S30 extract. After being programmed with 0.5 μg/mL of plasmid, protein synthesis reactions were carried out at 30 °C for 3 h. The amount of cell-free synthesized sf-GFP was determined by measuring the fluorescence intensity of 10-fold diluted reaction mixture in a VICTORTM X2 multilabel plate reader (PerkinElmer, Waltham, MA, USA). During the incubation of the reaction mixture, depending on the experiments, free ASODN or magnetic particles conjugated with ASODN or ASODN/RNase H were added to the reaction mixture to suppress protein synthesis. In the experiments for stop-and-go control of protein synthesis, addition and magnet-assisted removal of the conjugated magnetic particles were repeated. Throughout the experiments, SPSS Version 22.0 software (SPSS, Chicago, IL) was used for statistical analysis of data. Data with p-values smaller than 0.05 was considered to be statistically significant.

### Conjugation of oligodeoxyribonucleotides (ODNs) to magnetic particles

Magnetic particles modified with carboxyl group were used as the substrates for conjugation of oligonucleotides in this study. Surface carboxyl groups and the primary amine groups of the oligonucleotides were coupled by the EDC activation method (Supplementary Fig. 4a). Micro- or nano- magnetic particles, 1 mg suspended in 100 μL of 25 mM MES buffer (pH 6.0), were incubated with 5 nmole ODNs in the presence of 5 mg/mL EDC at room temperature for 30 min with gentle mixing. The unbound ODNs were removed by washing the magnetic particles with 300 μL of 50 mM Tris-HCl (pH 7.5) four times, after which the washed particles were stored in 1 mL of autoclaved DDW prior to use. The amounts of ODNs conjugated on the magnetic particles were quantified using the Qubit 2.0 Fluorometer (Invitrogen, Carlsbad, CA, USA).

### Co-conjugation of ODN and RNase H on nanoparticles for targeted degradation of sf-GFP mRNA

Co-conjugation of ASODN and RNase H on magnetic nanoparticles was conducted following two separate schemes using three oligonucleotides (see Supplementary Table 2). For the preparation of Type I particles, type I oligomer had the T_10_ linker sequence and the antisense sequence against target mRNA was modified with a 3′-amine group and a 5′-maleimide group. After being tethered on the carboxyl-modified nanoparticles through the 3′-amino group, the 5′-end of the oligonucleotide (150 pmole, when estimated after conjugation) was subsequently conjugated with RNase H by incubating the magnetic particles in 100 μL solution of 100 mM PBS buffer (pH 7.0) with 600 pmole of RNase H (Supplementary Fig. 4b). The resulting hybrid nanoparticles were washed with 200 μL of 50 mM Tris-HCl (pH 7.5) thrice and stored in the same buffer prior to use.

On the other hand, RNase H was conjugated with a separate linker oligonucleotide during the preparation of Type II hybrid nanoparticles. Type II oligomer-1, consisting of linker region and antisense region, was first conjugated on the EDC-activated nanoparticles through its 3′-end amine group and Type II oligomer-1 conjugated nanoparticles were washed with 200 μL of 50 mM Tris-HCl (pH 7.5) three times. To form a supporting stem structure with the linker region of the Type II oligomer-1, 4 times of Type II oligomer-2, carrying the 3′-maleimide group, was added to Type I conjugated nanoparticles and then placed at room temperature with gentle mixing after incubating of the mixture at 95 °C for 5 minutes. After washing the nanoparticles with 50 mM Tris-HCl (pH 7.5) to remove free ODNs, RNase H was coupled to the 3′-end of the Type II oligomer-2 following the same coupling chemistry used for the preparation of Type I nanoparticles. The amount of conjugated ODNs and RNase H was also determined using the Qubit 2.0 Fluorometer.

## Additional Information

**How to cite this article**: Lee, K.-H. *et al*. A molecular nanodevice for targeted degradation of mRNA during protein synthesis. *Sci. Rep.* **6**, 20733; doi: 10.1038/srep20733 (2016).

## Supplementary Material

Supplementary Information

## Figures and Tables

**Figure 1:**
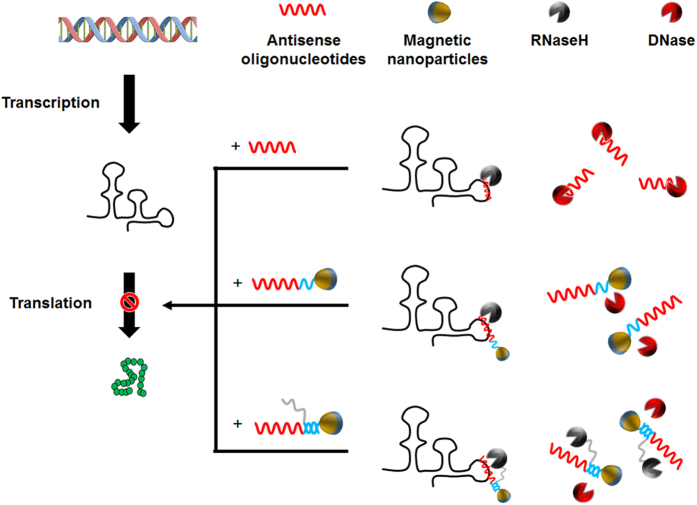
Three different approaches to study regulation of gene expression by ASODN. Free ASODN, ASODN-nanoparticle conjugate and ASODN/RNase H-nanoparticle conjugate were compared for their efficacy in targeted degradation of mRNA.
