# Supplementary material for: A molecular nanodevice for targeted degradation of mRNA during protein synthesis
Source: Sci Rep. 2016 Feb 9;6:20733. doi: 10.1038/srep20733 (PMC4746582; doi:10.1038/srep20733)
Supplement: Supplementary Information [file srep20733-s1.pdf]

Supporting Information

A molecular nanodevice for targeted degradation of mRNA during protein synthesis

Kyung-Ho Lee[1], Seung-Eui Min[1], Haseong Kim[2], Seung-Goo Lee[2] and Dong-Myung Kim[1,]*

[1]Department of Chemical Engineering and Applied Chemistry, Chungnam National University, Daejeon 305-764, Korea

[2]Synthetic Biology and Bioengineering Research Center, KRIBB, Daejeon 34141, Korea

Design and selection of ASODNs for the suppression of gene expression

We previously reported that the addition of appropriate ASODNs effectively repressed the expression of target genes during cell-free protein synthesis reactions.[1] Several candidate sequences of ASODN for sfGFP mRNA were selected using Sfold software.[2] The ASODN sequences were predicted to have high binding affinities between -13.0 and -16.0 kcal/mol and were tested for their efficiency in suppressing protein synthesis at 60 μM. Among the ASODNs examined, ASODN-3, which targets 20 nucleotides from positions 388 to 407, had the highest disruption energy and was most effective in the suppression of target gene expression. (ca. 94.8% suppression rate). Thus, we chose to use ASODN-3 in the subsequent experiments. To design a scrambled ASODN for ASODN-3, we used a software tool from GenScript Corporation (https://www.genscript.com/ssl-bin/app/scramble).

Effect of ASODN-MMP and ASODN-MNP on suppression of gene expression

Nanoparticles are polymeric particles in the nanometer size range, whereas microparticles are particles in the micrometer size range. Both types of magnetic particle can be used as vehicles to deliver ASODN to the reaction mixture of a cell-free protein synthesis system. We anticipated that the magnetic nanoparticle (MNP)-conjugated ASODNs would be more efficient than magnetic microparticle (MMP)-conjugated ASODSs in the suppression of gene expression due to their better accessibility to the large molecule of target mRNA. To compare two types of magnetic particle, MMP (1 μm in diameter) and MNP (50 nm in diameter) that were functionalized with carboxyl groups were conjugated with ASODNs labeled with an amine group at the 5'-end. We found that 4.56 nmol ASODN were conjugated per mg nanoparticles, whereas 1.36 nmol ASODN conjugated per mg microparticles. We compared antisense activity of different types of ASODN (free ASODN, ASODN-MMP and ASODN-MNP) by measuring the fluorescence intensity of cell-free translated sfGFP with ASODNs at a fixed final concentration of 10 μM.

Supplementary Table 1. Calculated sequences of ASODN candidates for targeting mRNA of sfGFP

Oligomers	Target position	Target sequence (5'->3')	Antisense oligo (5'->3')	GC content	Oligo binding energy (kcal/mol)
**ASODN-1**	256- 275	CGCUGGUGACCACCCUGACC	GGTCAGGGTGGTCACCAGCG	70.00%	-13.5
**ASODN-2**	257- 276	GCUGGUGACCACCCUGACCU	AGGTCAGGGTGGTCACCAGC	65.00%	-13.3
**ASODN-3**	388- 407	AUGGCAAAUAUAAAACGCGC	GCGCGTTTTATATTTGCCAT	40.00%	-15.5
**ASODN-4**	392- 411	CAAAUAUAAAACGCGCGCCG	CGGCGCGCGTTTTATATTTG	50.00%	-15.5
**ASODN-5**	393- 412	AAAUAUAAAACGCGCGCCGU	ACGGCGCGCGTTTTATATTT	45.00%	-15.5
**ASODN-6**	536- 555	UACGGCGGAUAAACAGAAAA	TTTTCTGTTTATCCGCCGTA	40.00%	-13.4
**ASODN-7**	537- 556	ACGGCGGAUAAACAGAAAAA	TTTTTCTGTTTATCCGCCGT	40.00%	-13.1
**Scrambled ASODN**	-	-	AGTTCGTCTTATACGTTGTC	40.00%	--

Supplementary Table 2. Sequence information of oligonucleotides for the conjugation of ASODN and RNase H to nanoparticles.

**Types of conjugate**	**Oligomers**	**Oligonucleotide sequences (5'→3')**
**Type I**	**Type I oligomer**	Maleimide-TTTTTTTTTTTGCGCGTTTTATATTTGCCA-Amine
**Type II**	**Type II oligomer-1**	GCGCGTTTTATATTTGCCATTTTTGGTGGTGCAGCTG-Amine
	**Type II oligomer-2**	CAGCTGCACCACCTTTTTTTTTTTTTT-Maleimide

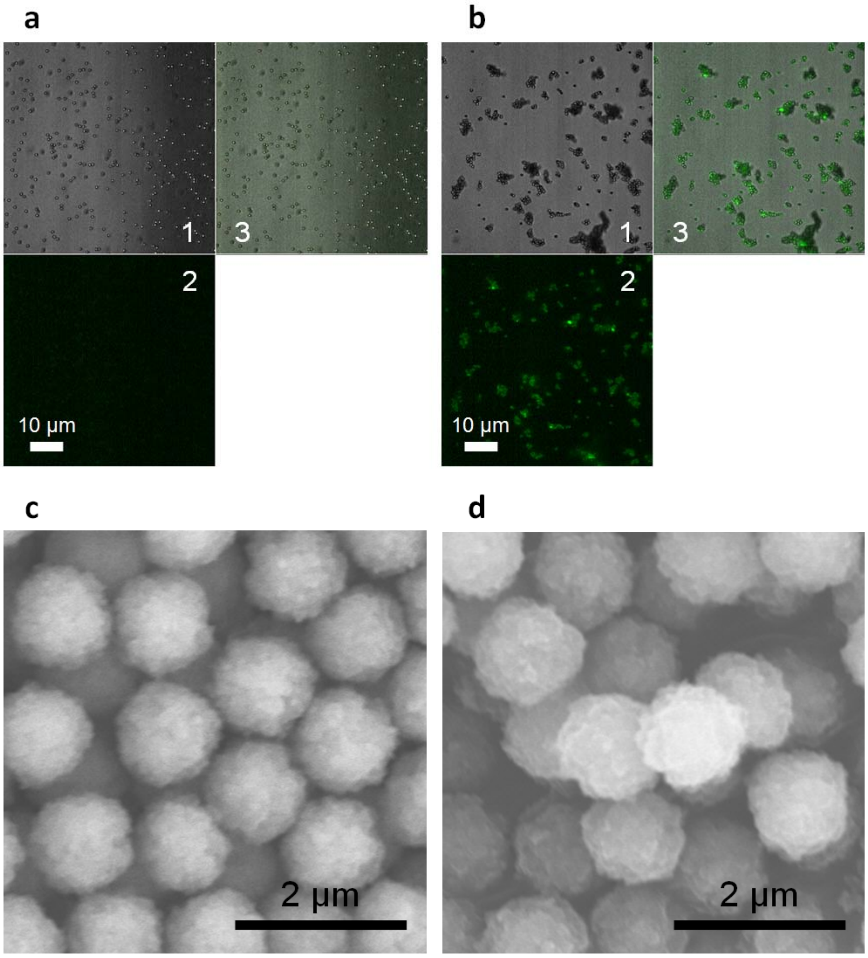

Supplementary Figure 1. Confocal laser microscopic images (a, b) and scanning electron microscope (SEM) images (c, d) of magnetic microparticles (1μm) before (a, c) and after (b, d) conjugation with ASODN labelled with FAM and amine at 5'- and 3'-end, respectively. 1, Optical images of the microparticles; 2, FAM fluorescence on the microparticles; 3, merged images of 1 and 2.

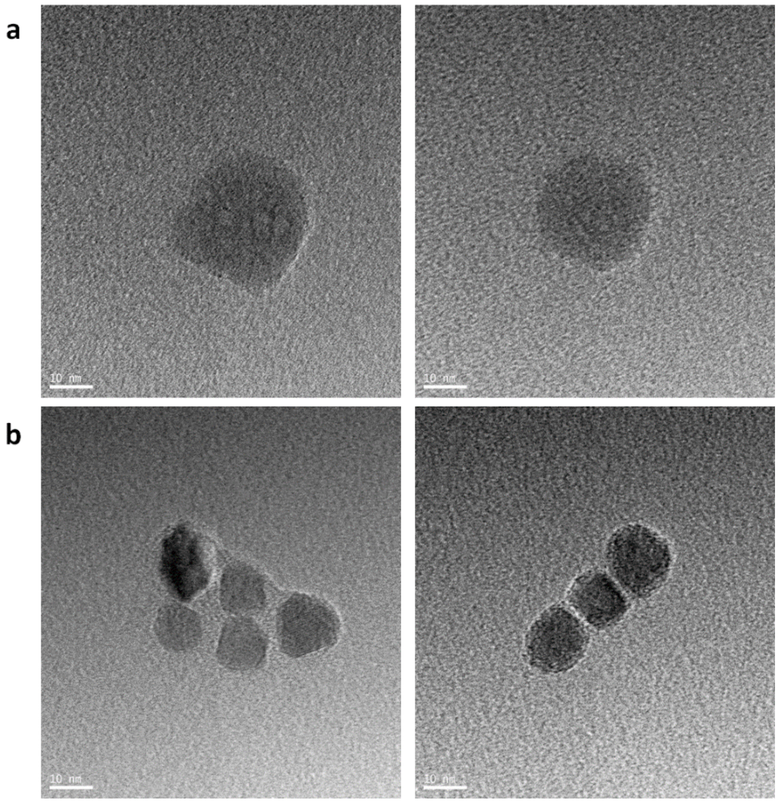

Supplementary Figure 2. Transmission electron microscopy (TEM) images of the magnetic nanoparticles before (a) and after (b) conjugation with ASODN.

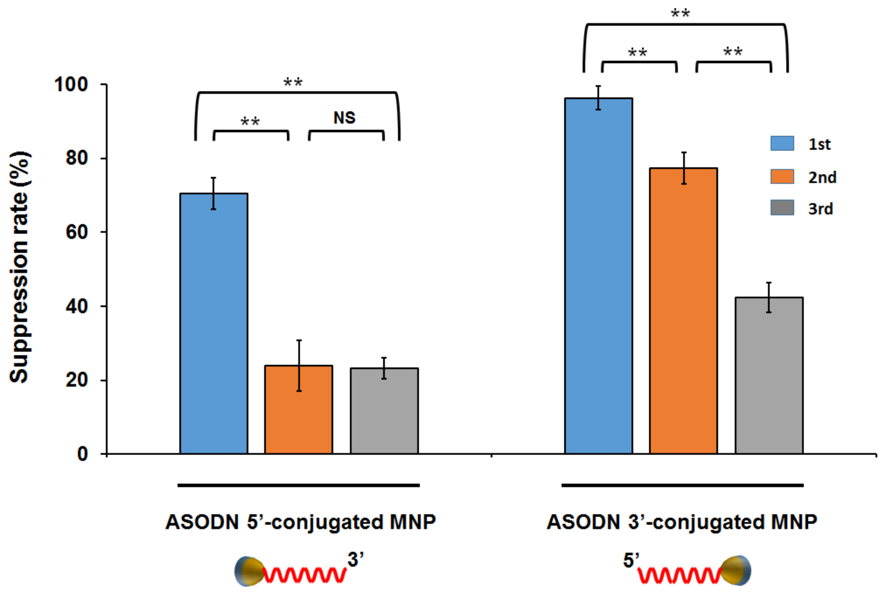

Supplementary Figure 3. Repeated use of ASODN-MNPs (10 μM) for the suppression of gene expression during cell-free synthesis reactions. Suppression rates are presented after the first (blue bars), second (orange bars) and third (gray bars) use of 5’- or 3’-conjugated ASODNs on MNPs. Error bars represent standard deviation of three independent reaction samples. ** p-values less than 0.01. NS, not significant.

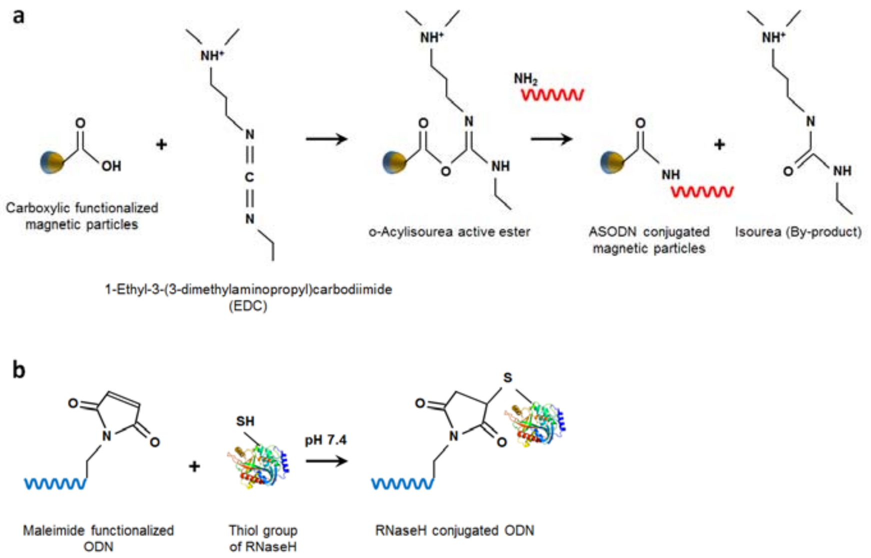

Supplementary Figure 4. Chemistries for conjugation of ASODN and RNase H on magnetic particles. (a) Conjugation of carboxyl functionalized magnetic particles with primary amine modified ASODN. (b) Conjugation of maleimide modified ODN with RNase H.

REFERENCES

1. Keum, J. W., Ahn, J. H., Kang, T. J. & Kim, D. M. Combinatorial, selective and reversible control of gene expression using oligodeoxynucleotides in a cell-free protein synthesis system. *Biotechnol. Bioeng.* **102**, 577-582 (2009).

2. Ding, Y., Chan, C. Y. & Lawrence, C. E. Sfold web server for statistical folding and rational design of nucleic acids. *Nucleic Acids Res.* **32**, W135-141 (2004).
